# Construction and Evaluation of Normalized cDNA Libraries Enriched with Full-Length Sequences for Rapid Discovery of New Genes from Sisal (*Agave sisalana* Perr.) Different Developmental Stages

**DOI:** 10.3390/ijms131013150

**Published:** 2012-10-12

**Authors:** Wen-Zhao Zhou, Yan-Mei Zhang, Jun-Ying Lu, Jun-Feng Li

**Affiliations:** South Subtropical Crops Research Institute, Chinese Academy of Tropical Agricultural Science, Zhanjiang 524091, China; E-Mails: yanmeizhang2002@163.com (Y.-M.Z.); junyinglusisal@163.com (J.-Y.L.); junfeng1467@163.com (J.-F.L.)

**Keywords:** sisal, normalization, full-length, cDNA library, *knox* gene, *MADS-box* gene, expression

## Abstract

To provide a resource of sisal-specific expressed sequence data and facilitate this powerful approach in new gene research, the preparation of normalized cDNA libraries enriched with full-length sequences is necessary. Four libraries were produced with RNA pooled from *Agave sisalana* multiple tissues to increase efficiency of normalization and maximize the number of independent genes by SMART™ method and the duplex-specific nuclease (DSN). This procedure kept the proportion of full-length cDNAs in the subtracted/normalized libraries and dramatically enhanced the discovery of new genes. Sequencing of 3875 cDNA clones of libraries revealed 3320 unigenes with an average insert length about 1.2 kb, indicating that the non-redundancy of libraries was about 85.7%. These unigene functions were predicted by comparing their sequences to functional domain databases and extensively annotated with Gene Ontology (GO) terms. Comparative analysis of sisal unigenes and other plant genomes revealed that four putative *MADS-box* genes and *knotted*-like homeobox (*knox*) gene were obtained from a total of 1162 full-length transcripts. Furthermore, real-time PCR showed that the characteristics of their transcripts mainly depended on the tight expression regulation of a number of genes during the leaf and flower development. Analysis of individual library sequence data indicated that the pooled-tissue approach was highly effective in discovering new genes and preparing libraries for efficient deep sequencing.

## 1. Introduction

As an important economic crop, Agave plants had been exploited and broadly utilized in different fields [1]. The best known modern agave products are tequila and agave sugars as dietary supplements and substitutes for sugar and fats [2,3] or for the production of paper [4]. Recently, some Agave species have been regarded as bioenergy crops [5] because their exploitation for bioenergy production will not divert resources from staple food crop production, as is the case of maize when used for bioenergy production [1]. Because Agave plants have many unique and interesting biological characteristics, they have also attracted the interest of plant physiologists, notably in the field of crassulacean acid metabolism (CAM) and their adaptation to arid climates, in addition to extensive studies by taxonomists [6,7]. Among the Agave species, *Agave sisalana* is one of the most important species in widespread cultivation and application [8].

Despite the current and potential economic importance and research interests, little basic research had been carried out on these species due to a lack of basic genetic knowledge, large genome size estimated at between 2940 and 4704 Mbp of DNA [9], and long life cycle (5–8 years), especially at the genetic and molecular levels. With comparable species such as pineapple [10], only ca. 310 sequences had been deposited for the whole Agave genus, including 82 sequences from *Agave tequilana*, which were mainly ribosomal genes, transposon-like sequences and chaperones. Although Simpson *et al.* had constructed *A. tequilana* cDNA libraries and got some sequence information [1], no large-scale genomic or transcriptomic sequencing data is available in Genbank.

Currently, genetic or breeding studies of intra- and interspecific crosses have been successful [11], but the conventional hybridization was at low efficiencies and high costs in terms of labor, resources and money. *In vitro* regeneration of most Agave species tested was relatively easy and could be achieved either by indirect organogenesis or through *in vitro* suspension culture [8,12]. However, the asexual propagation led to vulnerability in adverse environmental conditions or attack by pests and pathogens. Based on these reasons, exploring some functional genes will be critical for improving the physiological characterizes and breeding excellent varieties.

Construction of full-length cDNA library and sequencing of ESTs could help in rapid gene discovery, especially in non-model organisms where no prior sequencing data is available. Unfortunately, conventional cDNA libraries not only need high amounts of starting mRNA (5–100 μg), but also contain a high percentage of 5′ truncated clones due to the premature stop of reverse transcription (RT), especially large mRNAs tending to form secondary structures [13]. For these reasons, SMART™ technology for full-length enriched cDNA is very straightforward and robust and requires only 0.025–1 μg of starting mRNA [14]. This technology utilizes the property of some MMLV reverse transcriptases to add a few C residues at 3′ end of the first strand cDNA, but not at prematurely terminated reverse transcripts [15]. Moreover, the percentage of full-length clones with the SMART technique is much higher compared with other full-length enriching techniques [13,16].

Due to differences in the level of gene expression among various cell types, the construction of a normalized cDNA library is necessary in approximately equal quantities and substantially increasing the efficiency of the search for rare genes. However, most of the available approaches based on the re-association of amplified plasmid libraries are not appropriate for long cDNA normalization [17]. Interestingly, duplex-specific nuclease (DSN) from the hepatopancreas of the Kamchatka crab displays a strong preference for cleaving dsDNA and DNA in DNA-RNA hybrid duplexes compared with ssDNA and RNA, irrespective of sequence length. The use of this enzyme allows us to develop a new, simple and effective method for normalization of cDNA, enriched with full-length sequences [18].

To provide insight into transcription characteristics and rare gene functions involved in different development periods of *A. sisalana*, the objective of this study was to construct and characterize the normalized full-length cDNA library combining the robust SMART™ technique and DSN method. Sequences were evaluated and annotated to classify different categories. By contrast, *MADS-box* and *Knotted*1-like homeobox (KNOX) gene families were contained in our libraries. The *MADS-box* and *knox* genes had been demonstrated to regulate various aspects of development in all green plant lineages and play a key role in maintaining a pluripotent cell population, called the shoot apical meristem (SAM) [19–25]. Although only a few studies regarding these potential functional genes have been reported for Agave plants, there were no any references in *A. sisalana*. Here, we report on the isolation and functional analysis of one putative *Asknox* and four *MADS-box* genes positioned into three subclasses from sisal. These studies revealed that they could participate in regulating leaf formation and floral development, and helped us to understand how these proteins influence plant development.

## 2. Results and Discussion

### 2.1. Generation of the Full-Length Enriched and Normalized cDNA

By contrast, published protocols did not lead to equal representation among clones of different sizes, maintain the length of long cDNAs after hybridization, or incorporate simultaneous normalization and subtraction of cDNAs [14,15,26]. To avoid those problems related to amplification of libraries, exploring a technique to normalize and subtract cDNA before cloning was necessary. In our strategy, the first strand cDNA synthesis was performed in the presence of the SMART™ RNA oligo in the reaction. And then, the full-length selective step was carried out following cDNA synthesis. Because smaller cDNAs are more preferentially amplified than large cDNAs during PCR [13], more PCR cycles must be done on the large fractions to obtain an equivalent amount of PCR product for cloning. During our experiment operation, 14 cycles were adopted to avoid increasing redundancy and reduce errors introduced by PCR polymerase according to size fraction. By these means, large cDNAs could be amplified as efficiently as smaller ones. As a result, both the first-strand cDNA and the amplified cDNA turned out to be flanked by inverted terminal repeats, which can be applied later for both non-directional and directional cloning of cDNA libraries.

Based on the kinetics of cDNA re-association, DSN normalization differed from the other methods by a separation procedure of the normalized ss-fraction [18], and involved the denaturation and re-association of cDNA with the formation of a normalized ss-fraction and a non-target ds-fraction. In addition, DSN was a thermostable enzyme active at 70 °C, so the degradation of ds-fraction was carried out at the same temperature as the cDNA re-naturation. This helped avoid a nonspecific hybridization of cDNA during the DSN treatment, and the loss of transcripts prone to the formation of secondary structures [17]. This method has been applied to analyze mouse transcriptomes and demonstrated successful performance [27]. Normalization led to a 250-fold decrease in the representation of the high-abundant genes and brought the ca. 65%–70% frequency of full-length sequences [15].

### 2.2. Characterization of *A. sisalana* cDNA Libraries

**Figure 1:**
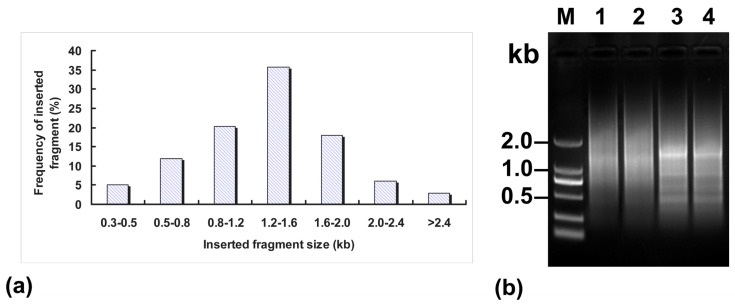
Features of normalized cDNA libraries enriched with full-length sequences. (**a**) Insert size distribution in normalized cDNA library; (**b**) Agarose gel electrophoresis of non-normalized and normalized amplified SMARTTM-prepared cDNA. Lane 1–2, normalized first-strand cDNA; lane 3–4, non-normalized cDNA; lane M, DL2000 ladder (Takara, Japan).

To rapidly discover some novel genes related to plant development, four normalized and full-length enriched cDNA libraries were constructed from different developing periods of *A. sisalana* tissues. The lengths and fullness ratios of cDNA inserts were investigated by PCR to assess the quality of those full-length enriched cDNA libraries [28]. As expected, most of the cDNA insert sizes ranged from 1 to 2 kb with an average length of 1.2 kb from normalized libraries (Figure 1a), which reflected the size distribution of the first-strand cDNAs (Figure 1b). However, no cDNAs longer than 3 kb were found in these samples. BlastX analysis of the sequences revealed that 35% (1162 out of 3320 unigenes) of the clones could potentially encode for full-length genes with an average length of 1.8 kb (Table 1). Redundancy rates were calculated in a clustering analysis of all ESTs generated from the normalized library using the program Megalign (Lasergene, DNAstar, Inc., Madison, WI, USA). In fact, the rate of recovery of unigenes in this study was about 85.6%, which is much higher than the 30% to 40% reported from non-normalized cDNA libraries [29,30]. This normalization will greatly help to enrich the library for rare genes, and increase the rate recovery of unigenes and reduce the cost of sequencing by avoiding redundant clones.

From four normalized and full-length enriched cDNA libraries, 4500 clones were randomly selected from the selection medium and sequenced using M13 reverse primer. Successful sequences were 3875. Those clones containing vector backbone and additional sequences that were added during cDNA synthesis were removed. The sequencing results were compared to genes in the non-redundant (nr) protein database using a BlastX search to determine the fullness ratios of the library (Table 1). Of these 3320 sequences, 2158 (65%) matched known genes, and 67% of the clones of classified known genes were predicted to contain a putative ATG translation initiation codon [13,16]. A total of 3320 putative novel transcripts included a large proportion of singletons (80.64%) and a small percentage of contigs (13.36%), which had no significant hits to the non-redundant (nr) protein databases of the NCBI. As expected, our libraries were abundant in several non-redundant and full-length sequences, which could be used for efficient deep sequencing in order to explore several rare genes.

### 2.3. Gene Ontology Annotation and Bioinformatics Analysis

**Figure 2:**
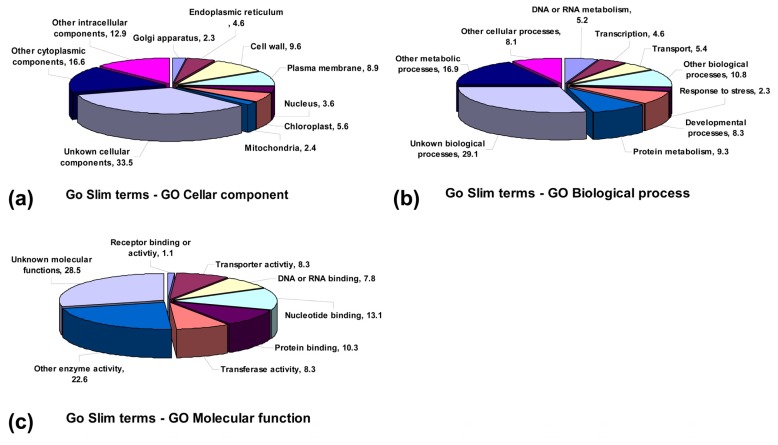
Functional categorization of unigenes with Gene Ontology (GO) terms at The Arabidopsis Information Resource (TAIR). These results in the unigenes were functionally classified under three main functional categories: cellular component (**a**), molecular function (**b**) and biological process (**c**) with respective GO Slim terms.

In our study, contig assembly was done to remove the redundant ESTs and produce a set of unique, high-fidelity virtual transcripts (unigenes). The partial full-length unigenes were compared to these protein sequences published in the databases using the Blastx program (Table 2). Based on comparation and pfam annotations, *A. sisalana* unigenes were further annotated for each unigene with Gene Ontology (GO) terms. A total of 3320 unigenes were assigned in the biological process category, molecular function category and cellular component category (Figure 2). Most of the terms in which the *E*-value showed significance were enriched in our unique sequences. This classification provided information on the percentage of unigenes involved in the signal transduction, anabolism, catabolism, reproduction etc. Based on their function in different cellular compartments and anatomical structures, the majority of the unigenes were grouped under “other intra-cellular components”, “unknown cellular components”, and “other cytoplasmic components”, which accounted for about 63% of the unigenes (Figure 2a).

In contrast to their biological functions, the unigenes were then classified into nine different metabolism processes and an unknown item. The larger part of the unigenes were divided into “other cellular process”, “other biological process”, “other metabolic process” and “unknown biological processes” accounting for 8.1%, 10.8%, 16.9% and 29.1%, respectively (Figure 2b). In addition, a large number of *A. sisalana* unigenes appeared to be involved in plant molecular functions, such as transporter activity, kinase activity, transferase activity, or nucleotide and protein binding activities (Figure 2c). Of the unique sequences, it was indicated that the normalization and identification of new functional genes from our full-length cDNAs were very efficient.

### 2.4. Tissue-Specific Gene Expression

#### 2.4.1. *Asknox* Expression Characteristics Related to Organogenesis

**Figure 3:**
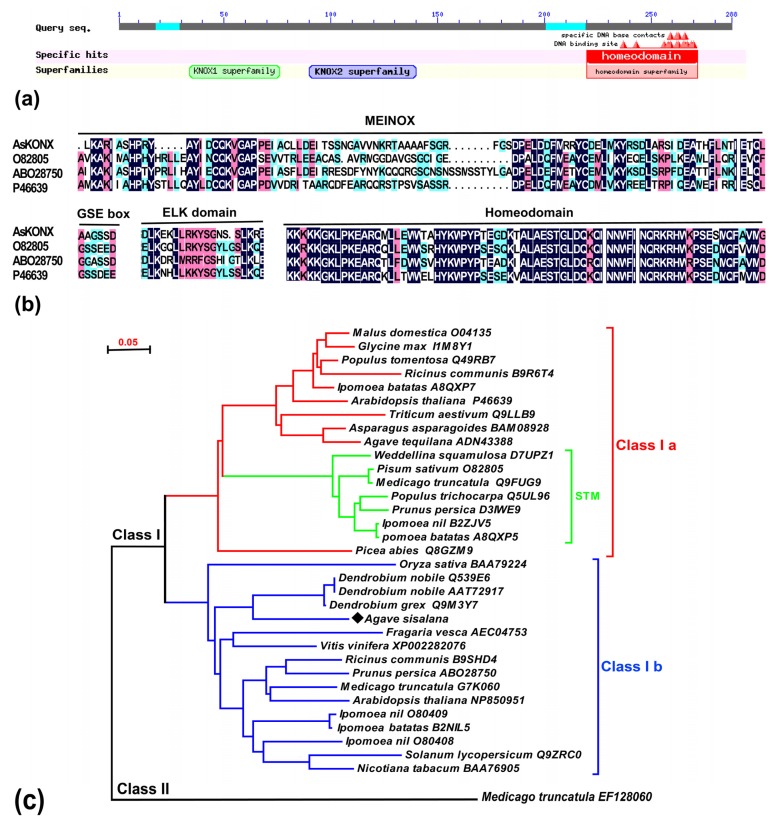
Sequence alignment and phylogenetic tree of the AsKNOX protein selected relatives from other plant species. (**a**) Schematic diagram of the AsKNOX protein structure by Position-Specific Iterated Blast; (**b**) Alignment of the deduced amino acid sequences of AsKNOX and KNOXs from *A. thaliana* (P46639), *Pisum sativum* (O82805) and *Prunus persica* (ABO28750). Identical and conserved amino acid residues were labeled in various colors, respectively. The different functional domains were indicated by black lines above the corresponding sequences. Dashes indicated gaps introduced to optimize the alignment. Sequences were aligned using the CLUSTALX program of DNAman; (**c**) Relationship tree of AsKNOX and its relatives in the class I KNOX group. The tree was obtained using the neighbor-joining method performed with the amino acid sequence alignment of the region comprising the MEINOX, GSE and HD domains. To assess support for the inferred relationships, 1000 bootstrap samples were generated. Sequences were designated by accession numbers and the name of organisms from the GenBank database. One Class II KNOX protein from *M. truncatula* was used as outgroup.

Plant leaves develop as flat lateral organs from SAM, and the establishment of polarity along three-dimensional axes (the proximodistal, mediolateral and adaxial-abaxial axes) is crucial for the growth of normal leaves [20], especially for sisal. In our study, a *knotted*-like homeobox (*knox*) gene (*Asknox*, 1074 bp) encoded homeodomain-containing transcription factor was obtained from the early developmental cDNA library of *A. sisalana*. Homology comparison, AsKNOX protein sequence shares 57% with *A. thaliana* KNAT2 and KNAT6 (ID: NP850951), 53% identity with *Solanum lycopersicum* TKN (ID: Q9ZRC0), and 51% with closely-related monocot genes, *O. sativa* OSH6 (ID: BAA79224). As with other species, AsKNOX possesses all three highly conserved domains typical to the KNOX proteins: the MEINOX domain that is subdivided into KNOX1 and KNOX2 (Figure 3a), the ELK domain and the homeodomain (Figure 3b). AsKNOX also contained a GSE domain between the MEINOX and ELK domains, similar to previous reports [31]. It is therefore reasonable to assume that AsKNOX protein possesses DNA-binding activity via its homeodomain, dimerization activity via its MEINOX domain, a nuclear localization signal via its ELK domain, and a protein degradation signal via its GSE domain [31,32].

A phylogenetic comparison of class I KNOX proteins, including that of *A. sisalana*, using amino acid sequences covering the MEINOX-HD region, enables the subdivision of the protein family into two main clades, namely Classes Ia and Ib (Figure 3c). The global tree topology reveals that KNOX protein sequences from diverse species positioned AsKNOX with class Ib KNOX proteins. Though monocots and dicots are all found in two subclasses, distinct boundaries can be distinguished between them (Figure 3c). On the basis of these relationships, it can be postulated that at least one duplication in the ancestral class I gene occurred before the monocot-eudicot split, leading to the divergence of class Ia and class Ib groups of genes [33].

**Figure 4:**
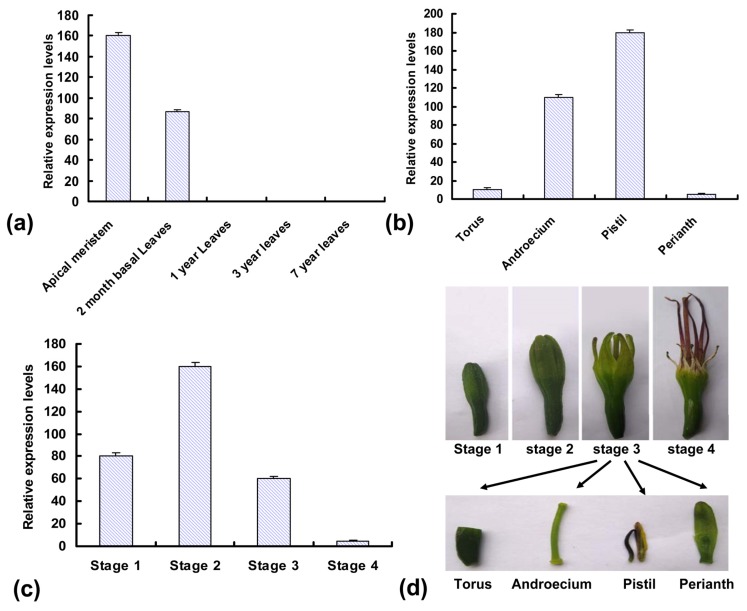
Real-time PCR analysis of *Asknox* gene expression profiles in different leaf and inflorescence development stages. (**a**) Analysis of the *Asknox* expression profiles in apical meristem and leaves during different development stages; (**b**) Analysis of the *Asknox* expression profiles in different development stages of inflorescence; (**c**) Analysis of the *Asknox* expression profiles in different organs of inflorescence; (**d**) Schematic diagram of different development stages and organs of inflorescence.

To understand transcription characteristics of *Asknox*, real-time PCR was performed in different development stages of leaf and flower. *Asknox* transcripts were mainly expressed in the vegetative shoot apex containing the single SAM and also in the developing inflorescence (Figure 4). Interestingly, *Asknox* was strongly expressed in the apical meristem and at relative low levels in leaf tissues, and could not be detected in grown-up leaves with analogous expression reports of KNOX family genes in other species [20,22]. However, the transcription expression is still detectable in early developing leaves (Figure 4a). It was postulated that Class I *Asknox* expression might correlate with sisal leaf shape, like simple-leafed species, such as *A. thaliana* and maize [34,35]. Ten years after *knox* overexpression was first shown to increase tomato leaf complexity, genetic evidence was provided that KNOX activity was necessary and sufficient for leaflet formation in *C. hirsuta* [36]. A similar explanation had been offered for the variable leaf phenotypes observed in transgenic tobacco plants constitutively expressing the tobacco *konx* genes, *TKN1* and *TKN2* [37]. Loss-of-function and over-expression of *knox* genes had serious effects on shapes and sizes of leaves. The reason is that KNOX proteins may regulate hormone levels and repress the transcription of the IAA and GA-synthetic genes, and have impressive effects on leaf morphology [38,39]. These results suggested that *knox* transcription factors drive leaf development in various ways in different species by controlling the temporal action of cellular growth and differentiation pathways during early stages of the leaf.

In addition, *Asknox* transcripts were also readily detectable in early developing floral buds with high levels in pistil and androecium, but barely detectable in torus and perianth (Figure 4b). *Asknox* expression was increased 2-fold in developing floral buds with early initiating petal-spur primordial (Figure 4c), and supports the hypothesis that *Asknox* may also have a role in keeping normal patterning in these tissues. This hypothesis was identified by analysis of transgenic plants in Arabidopsis and strawberry, where defects in flower form, petal abscission, fruit set, and fertility were observed [25]. Thus along with leaf development, *Asknox* plays a significant role in floral architecture and function, consistent with it relatively high level of expression in these tissues.

#### 2.4.2. Expression Characteristics of *MADS-Box* Gene Family Related to Floral Development

**Figure 5:**
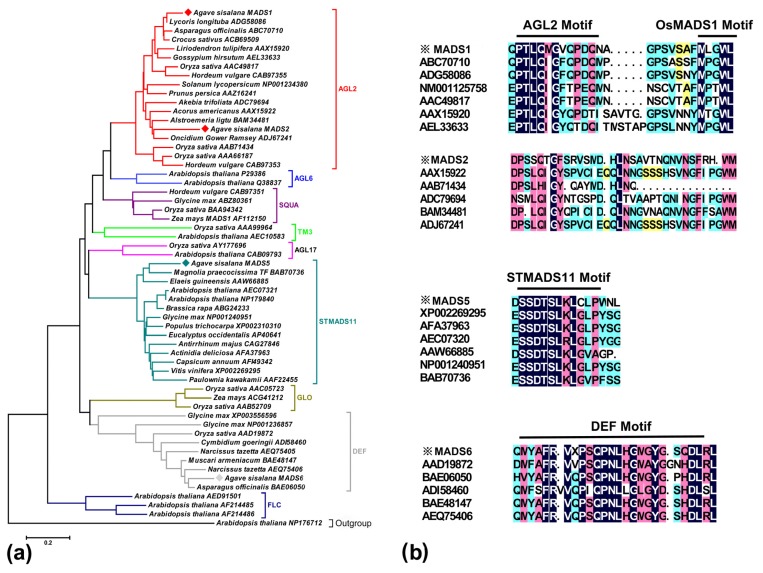
Phylogenetic tree and *C*-terminal domain alignment of the deduced *MADS-box* protein sequences from *A. sisalana*. The neighbor-joining tree had been constructed using a representative subset of 58 sequences from available plant *MADS-box* sequences (**a**). These 58 sequences have been selected as follows: To assess support for the inferred relationships, 1000 bootstrap replications were generated. Alignment of *C*-terminal amino acid domains of *A. sisalana* MADS proteins (**b**) based on motifs previously identified by Vandenbussche *et al.* [41]. Sequences are designated by accession numbers and organism names from GenBank database.

A search for gene sequences in the *A. sisalana* transcriptome database, four potentially distinct *MADS* genes (*AsMADS-box1*, *2*, *5* and *6*) including the putative complete open reading frames were determined. The alignment analysis showed that *AsMADS-box1*, *2*, *5* and *6* shared with amino acid identities of 90%, 84%, 82% and 91% to *MADS-box1* (AEX92976), *MADS-box2* (AEX92975), *MADS-box5* (AEX92969) and *MADS-box6* (AEX92972) from *A. tequilana*, respectively (Supplementary data, Figure S1). As most plant *MADS-box* sequences, four homology proteins from *A. sisalana* fell into the stereotypical type II category on the basis of the full-length amino acid sequences. Further analysis showed that four genes belonged to three subfamilies, such as AGL2, STMADS11 and DEF, respectively (Table 3, Figure 5a). Highly conserved amino-acid consensus sequences were also found in their *MADS-box* domains (Figure 5b). A coiled-coil structure (*K* domain) also appeared. In addition, the *MADS-box* and *K* domains were separated by a weakly conserved intervening domain (*I* domain) (data not shown). It had been shown that the *I* and *K* domains were involved in protein-protein interactions [40].

**Figure 6:**
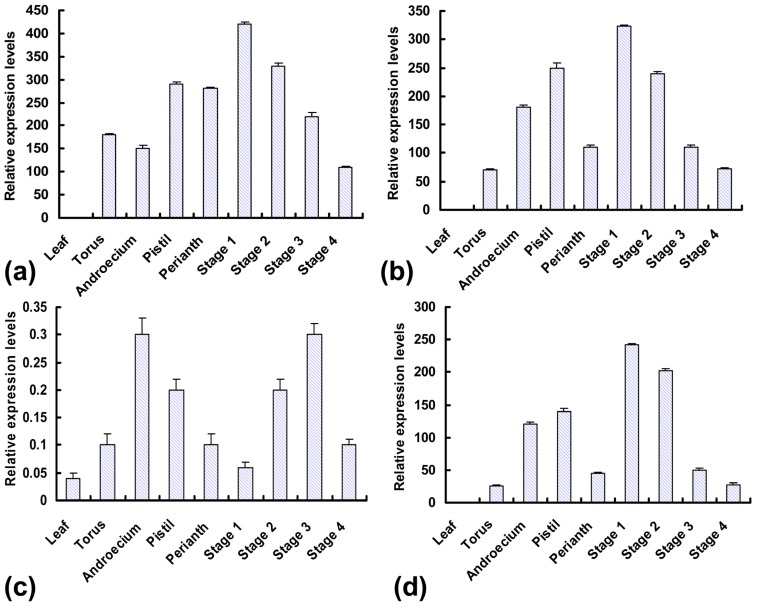
Real-time PCR analysis of *AsMADS-box* gene expression profiles in different tissues and inflorescence development stages. (**a**) *AsMADS-box1* gene expression profiles; (**b**) *AsMADS-box2* gene expression profiles; (**c**) *AsMADS-box5* gene expression profiles; (**d**) *AsMADS-box6* gene expression profiles.

Although the *MADS-box* genes had been extensively investigated and shown to be essential for inflorescence and flower development in many model plants [23,24], there was an infrequent research in *A. sisalana*. On the basis of protein sequence alignment, *AsMADS-box1* and *2* were closely related to *SEPALLATA3* (*SEP3*) and *SEPALLATA2* (*SEP2*) within the E function genes, which were required for the formation of petal, stamen and carpel [19]. Interestingly, in our study, real-time PCR revealed that the parallel expression patterns showed between the *AsMADS-box1* and *2*, although *AsMADS-box1* had more highly expressed levels compared with *AsMADS-box2* (Figure 6a, b). In inflorescence tissues, the high levels of expression were observed for *MADS-box1* and *2* at the initial stages of bud development and decreased during flower formation; however, no expression was detected in fully developed leaf tissue. These results were similar to *SEP*-like and *OsMADS1*-like gene expression patterns in orchid and *A. tequilana*, which were detected in inflorescences and developing flowers [42,43]. This was predicted that they might encode proteins of redundant functions based on homology analysis with Arabidopsis [44]. The significant fold changes of expression levels between *MADS-box1* and *2* of E function genes suggested that *MADS-box1* need to be further analyzed in order to demonstrate the role in the floral transition and development in *A. sisalana*, and may also reflect the diversity in the E group genes in various plant species during evolution.

In addition, interactions between the proteins encoded by the ABC type genes and the SEP-like genes were essential for the correct regulation of flower development. This assumption was further supported by the interaction between SEP and B, C function proteins [45]. For example, AP3 and PI had been shown to regulate petal and stamen development by interacting with SEP1, SEP2, and SEP3 [19,45,46]. This result strongly indicated that E function genes were necessary for the activities of the B and C function genes.

However, *MADS-box5* showed no significant differences in expression levels between any flower organs and developmental stages (Figure 6c), because *StMADS11*-like genes had been shown to play an important role in the vegetative to floral transition as in the case of SVP from *A. thaliana* that repressed the switch from vegetative to floral growth [47,48]. The *svp* mutants of *A. thaliana* flowered earlier due to a reduced vegetative growth phase, and also passed more rapidly during the different stages of vegetative growth [49].

Based on the pattern of *MADS-box6* (*DEF*-like) expression with similar expression profiles to *AsMADS-box2* gene, it was conceivable that this gene could also exert some effect on flower formation (Figure 6d). In *A. thaliana*, heterodimers form between DEF and GLO proteins, which in turn interact with SEP-like proteins to form a functional unit. Similar homodimerization was also found in tulip [50], although the functionality of these complexes in specific floral organs was unclear. Sandoval *et al.* reported that the *A. tequilana DEF*-like and *GLO*-like genes show disproportionate changes in expression levels due to homodimerization formation of DEF-like protein [43]. In *A. sisalana*, whether the heterodimerization and homodimerization are produced between AsMADS and GLO-like proteins and interact with SEP-like proteins still need to be further investigated.

The data presented here show that MADS factors play different roles in the developmental pathway that finally leads to plant reproduction. Due to the complex genetic background in *A. sisalana*, how the *MADS-box* genes regulate the diverse developmental processes ranging from root to flower and fruit development is still further explored using the mutated and transgenic plants. With the gene functions elaborated in some exciting models and the novel genes discovered using the normalized cDNA library, those will considerably contribute to a better understanding of the relationship between functional genes and plant development.

## 3. Experimental Section

### 3.1. Plant Materials

Field-grown sisals (*Agave sisalana* Perr.) were obtained from the Sisal Field Germplasm Bank of the Southern Subtropical Crop Research Institute of CATAS (Guangdong, China). Four development stages of different tissues (SAM, root, stem, flower, and leaf) were randomly sampled from two month seedlings in the greenhouse to one, three, and seven year plants in the field. These sliced tissues of three independent plants were pooled together as one of three replications at each sampling stage. All of the tissues were immediately frozen in liquid nitrogen and stored at −80 °C before being analyzed.

### 3.2. Poly(A^+^) RNA Isolation and First-Strand cDNA Synthesis

Total RNAs isolated from different tissues using Trizol reagent (Life Technologies Inc., Invitrogen, Carlsbad, CA, USA) were assessed by absorbance at 260 nm and 280 nm and agarose gel electrophoresis (1.5%), respectively. For construction of those full-length enriched cDNA libraries, SMART™ PCR cDNA Synthesis Kit (BD Biosciences Clontech, San Jose, CA, USA) was used for the synthesis of cDNA starting from 0.5 to 1 μg of poly (A^+^) RNA according to the manufacturer’s instruction. The primers were the BD Biosciences oligonucleotides SMART™ Oligo VI and CDS-3M containing the *Sfi*I A and *Sfi*I B recognition sequence, respectively (Supplementary data, Table S1).

### 3.3. Amplification of cDNA by Long-Distance Polymerase Chain Reaction

To prepare the full-length normalized cDNA, the first strand cDNA was amplified with PCR primer provided in the SMART™ PCR cDNA Synthesis Kit (Clontech). The PCR mixture (50 μL) contained 1 × Advantage 2 Polymerize mix, 1 × Advantage 2 PCR reaction buffer, 200 mM dNTPs, 0.3 mM primer and 3 ng first-strand cDNA. Fourteen PCR cycles (95 °C for 7 s, 65 °C for 20 s, and 72 °C for 3 min) were performed. The samples were used for the DSN normalization and the preparation of cDNA libraries.

### 3.4. First-Strand cDNA Normalization and Amplification

Upon completion of first-strand cDNA synthesis, the reaction mixture was purified using the Qia Quick PCR Purification Kit (Qiagen, Tokyo, Japan), and dissolved in milliQ water to a final cDNA concentration of 100 ng/μL after precipitation with ethanol. The reaction mixture (1.5 μL aliquot, 200 mM Hepes, pH 7.5, 2 M NaCl, and 0.8 mM EDTA) was denatured at 98 °C for 3 min and allowed to hybridize at 70 °C for 5 h. After re-naturation, 5 μL of 2×DSN buffer (100 mM Tris-HCl pH 8.0, 10 mM MgCl_2_, and 2 mM dithiothreitol), preliminarily preheated to 70 °C, was added to cDNA samples for 10 min. Then, 0.25 Kunitz units of DSN enzyme (Evrogen, Russia) were added to the reaction mixture for 20 min at 70 °C, and were subsequently inactivated by the addition of 10 μL of 5 mM EDTA.

To amplify the normalized ssDNA fraction after DSN treatment, PCR was carried out in a 50 μL reaction mixture using an advantage 2 PCR kit (BD Biosciences Clontech, United States) containing 2 μL of reaction mixture, 1 × Advantage 2 Polymerize mix (Clontech), 1 × Advantage 2 PCR reaction buffer (Clontech), 200 mM dNTPs and 0.3 mM CapM primer, which corresponds to the external part of the flanking cDNA adapter. To obtain amplified cDNA samples with a concentration of ca. 20 ng/μL, twenty PCR cycles (95 °C for 7 s, 65 °C for 20 s, and 72 °C for 3 min) were performed.

### 3.5. Construction and Quality Analysis of cDNA Library

Normalized cDNA samples digested by *Sfi*I were cloned into the pDNR-LIB vector (Promega Corp., Madison, WI, USA) with T4 DNA liganse (MBI Ferments Inc., Vilnius, Lithuania), which were used for *Escherichia coli* (JM109) electro-transformation with the Gene Pulser II system (Bio-Rad company, Richmond, CA, USA) at 1500 V. After shaking for 1 h at 120 rpm and 37 °C, 200 μL of the electroporated cell were spread on Luria-Bertani (LB) agar plate containing 30 μg/mL of chloramphenicol. Frequencies of the corresponding cDNA sequences in the libraries were calculated from the number of positive colonies. For analysis of the insert size distribution, 200 colonies from each library were randomly picked, and were used for PCR with standard M13 primers (Supplementary data, Table 1). The reaction system was followed by denaturation at 94 °C for 10 min, 30 cycles (94 °C for 30 s, 52 °C for 30 s, and 72 °C for 2 min), and 72 °C 10 min. PCR products were visualized on a 1.2% agarose gel, following ethidium bromide (EB, 0.5 pg/mL) staining, alongside a 1 kb DNA ladder (Takara, Japan).

### 3.6. Sequence Processing and Analysis

Vector-derived and ambiguous sequences were eliminated using online software (vecscreen) from NCBI. The EST sequences were clustered and assembled into contiguous consensus sequences (contigs) using the program Seqman and Megalign of DNAstar software. The non-redundant sequences were searched against protein databases obtained from NCBI with a search threshold of *E* < 1.0 × 10^−5^. All similarity searches were executed using the BlastN, BlastX, and tBlastX tools. Phylogenetic trees were constructed by the Neighbor-Joining (NJ) method using the NJ algorithm implemented in the Molecular Evolutionary Genetics Analysis (MEGA) software version 5.0. The Blast results were used to obtain further information on the function and motif through the InterPro member databases [51].

### 3.7. Gene Expression Analysis by Real-Time PCR

Quantitative PCR was performed by using the first strand cDNA as templates on a Lightcycler (Roche Diagnostics), with the Light Cycler Fast Start Reaction Mix MasterPLUS SYBR Green according to the manufacturer’s recommendations. Cycling conditions were as follow: 95 °C for 5 min, 40 cycles at 95 °C for 10 s, 54 °C for 30 s, and 72 °C for 30 s. Expression of 18 s rRNA was used as an internal control to normalize the amount of mRNA. The data shown represent means of values obtained from three independent biological replicates.

## 4. Conclusions

Four sisal cDNA libraries enriched with full-length sequences were constructed by uniting SMART™ technique and the duplex-specific nuclease (DSN). Sequencing of 3875 cDNA clones revealed 3320 unigenes (85.7%) with an average insert length about 1.2 kb, which were extensively annotated with Gene Ontology (GO) terms. This DSN normalization greatly helped to enrich the library for rare genes and increase the rate recovery of unigenes. Furthermore, real-time PCR showed that the transcript characteristics of four putative *MADS-box* genes and one *knotted*-like homeobox (*knox*) gene mainly depended on the tight expression regulation of a number of genes during the flower and leaf development, respectively. Comparative analysis revealed that the pooled-tissue approach was highly effective in discovering new genes and preparing libraries for efficient deep sequencing.

## Supplementary Materials



## Figures and Tables

**Table 1:** Summary of cDNA sisal libraries.

Description	Number
Total number of successful sequences	3875
Unique sequences	3320
5′ end unigenes	2080
3′ end unigenes	2402
Full-length genes	1162
Number of known genes	2158
Unique unknown genes	1162

**Table 2:** Putative functions assigned to unigenes from sisal normalized libraries.

Library ID a	Functional annotation b	*E*-value	Accession No.	Organism species
SF1324	*Knotted*1-like homeobox protein	4 × 10^−112^	CAB88029	*Dendrobium nobile*
SL980	NADP-dependent malic enzyme	1 × 10^−91^	ABR26037	*Oryza sativa*
SL712	cytochrome P450 like_TBP	3 × 10^−95^	BAA10929	*Nicotiana tabacum*
SL349	ATP synthase subunit beta	6 × 10^−122^	XP003627732	*Medicago truncatula*
SF1073	unknown protein	2 × 10^−133^	ACU14517	*Glycine max*
SF1130	*MADS-box* transcription factor	4 × 10^−155^	ABC70707	*Asparagus virgatus*
SL453	chloroplast photosystem	1 × 10^−37^	ACZ54010.1	*Wolffia arrhiza*
SL324	Sucrose synthase	8 × 10^−104^	XP003591492	*Medicago truncatula*
SS239	Disease resistance protein	2 × 10^−45^	XP002275269	*Vitis vinifera*
SS136	UDP-glucosyltransferase	2 × 10^−80^	NP001154307	*Arabidopsis thaliana*
SF201	Ribulose-1,5-bisphosphate carboxylase	3 × 10^−180^	AFA55129	*Agave schottii*
SS712	Conserved hypothetical protein	2 × 10^−110^	BAD94036	*Arabidopsis thaliana*
SL506	Methionyl-tRNA synthetase	4 × 10^−189^	AAC99620	*Oryza sativa*
SF321	Sugar transporter	2 × 10^−98^	CAA90628	*Arabidopsis thaliana*
SF603	Poly(A)-binding protein	4 × 10^−110^	CAC01238	*plumbaginifolia*

a : The library ID is indicated by the first letter of the designation: S, sisal and the second letter of the designation: S, stem; L, leaf; F, flower.

b : Similarity gene function was conducted using the BLASTX program.

**Table 3:** Functional prediction of *MADS-box* genes from *A. sisalana* compared with Arabidopsis.

Gene name	Orthologous gene a	*E*-value	Max identity	Gene subfamily	Functional role predicted
*MADS-box1*	NP_564214 (SEPALLATA 3)	5 × 10^−108^	67%	AGL2	E function
*MADS-box2*	NP_186880 (SEPALLATA 2)	1 × 10^−82^	60%	AGL2	E function
*MADS-box5*	NP_179840 (SVP)	2 × 10^−90^	61%	STMADS11	Control of flowering time
*MADS-box6*	NP_191002 (APETALA 3)	7 × 10^−69^	51%	DEF	Class B floral homeotic gene

a : The classification was based on the Arabidopsis transcription database [40].
