# Supplementary material for: Construction and Evaluation of Normalized cDNA Libraries Enriched with Full-Length Sequences for Rapid Discovery of New Genes from Sisal (Agave sisalana Perr.) Different Developmental Stages
Source: Int J Mol Sci. 2012 Oct 12;13(10):13150–68. doi: 10.3390/ijms131013150 (PMC3497318; doi:10.3390/ijms131013150)
Supplement: Supplementary file 1 [file ijms-13-13150-s001.pdf]

Supplementary Information

Table S1. Oligonucleotide primers used for normalized cDNA construction, PCR detection and gene expression analysis (*N* = A, C, G, or T; *K* = A, G, or C).

Primer name	Primer sequence (5' to 3')
SMART oligo IV	AAGCAGTGGTATCAACGCAGAGTGGCCATTACGGCCrGrGrG
CDS-3M adapter	AAGCAGTGGTATCAACGCAGAGTGGCCGAGGCGGCC(T)20 KN
CapM primer	AAGCAGTGGTATCAACGCAGAGT
M13	TGTAAAACGACGGCCAGT
	CAGGAAACAGCTATGACC
*AsMDAS-box*1	GCTGCAGAGTAGTCATCAAGAGT
	GACTGTGCAAGGTATCCATTTGT
*AsMDAS-box*2	GGTCGCCCTCATCATCTTCT
	GCCGGCTGTATCCCACCATT
*AsMDAS-box*5	GCTCAAGTACGAAGGAGATTATTG
	CACGACTTAATCCAGTTTCAAGAG
*AsMDAS-box*6	GCCCTCGTCATCTTCTCCAACC
	GCCGGCTGTATCCCACCATT
18S rRNA	GGCCTTCGGGATCGGAGTAAT
	CTAAGAACGGCCATGCACCAC
AsKNOX	CCGCCGACGACCTCCACAC
	GATCCCCACTTTCGCCTTTA

Figure S1. Alignment of *MADS-box* amino acid sequences between *A. sisalana* and *A. tequilana*.

```
MADS-box_1.pro   MVGRGRVELKRI ENKI NRAVTFAKDRNGLNKKAYELSVLCDAEVALVI FSNRSKLYEFCSS. SSMMKTLERYQKCSYGAP   79
AEX92976.pro     MVGRGRVELKRI ENKI NRQVTFAKRRNGLLKKAYELSVLCDAEVALVI FSNRGKLYEFCSS. SSMMKTLERYQKCSYGAP   79
MADS-box_2.pro   MVGRGRVELKRI ENKI NSQVSFAKRRNSLLKKAYELSVNCDAEVALI I FSSRGRLFEFCSS. SSMDKTLEVYQNSSNNTL   79
AEX92975.pro     MVGRGRVELKRI ENKI NRQVSFAKRRNGLLKKAYELSVLCDAEVALI I FSSRGRLFEFCSS. SSMFKTLERYQNSSNNTL   79
MADS-box_6.pro   MVGRGKI EI KKI ENPTNRQVTYSKDRSGI MAKAKEATVLCDAETSLVMFSSAGKFSEYCDPSTDTKKSFDRYQQATG. . I   78
AEX92972.pro     MVGRGKI EI KKI ENPTNRQVTYSKRRSGI MKKAKELTVLCDAEVSLVMFSSTGKFSEYCSPGTDTKKI FDRYQQATG. . I   78
MADS-box_5.pro   MA. REKI NI RKI DNTTARQVTFSKRRRGLNKKAEELSI LCDAEVGLI I FSATGKLFEFSSS. . STKEI I EDHSMHSKKI L   77
AEX92969.pro     MA. REKI NI RKI DNTTARQVTFSKRRRGLFKKAEELSI LCDAEVGLI I FSATGKLFEFSSS. . STKEI I ERHSMHSKKI L   77

MADS-box_1.pro   DNSVQI RENQMLQSSHQEYLKLKARVEALQRSQRNLLGNDLGPLSSKELEQLERQLDSSLKQI RSTRTQYMLDQLADLQR   159
AEX92976.pro     DNSVQI RENQMLQSSHQEYLKLKARVEALQRSQRNLLGEDLGPLSSKELEQLERQLDSSLKQI RSTRTQYMLDQLADLQR   159
MADS-box_2.pro   KAMASSKET. . . QNSNEEYLKLSARFELLQLSQRNLLGEDLGQLSSNELEQLESQLAMSLKQI RSSKTQNMLGQLCDLKR   156
AEX92975.pro     KAMASSKET. . . QNSYEEYLKLKARFELLQLSQRNLLGEDLGQLSSNELEQLESQLEMSLKQI RSSKTQNMLGQLCDLKR   156
MADS-box_6.pro   NLWTAQYEK. . . . . MQNTLNHLKEI NYNLRKEI RQRMGEELDGMDVKDLRGLEQNLDEALKLVRHRKYHVI TTQTETYKK   153
AEX92972.pro     NLWTAQYEK. . . . . MQNTLNHLKEI NYNLRKEI RQRMGEELDGMDVKDLRGLEQNLDEALKLVRHRKYHVI TTQTETYKK   153
MADS-box_5.pro   SPEQPSLDLN. . . LQNDNYARLSKQVDETSRQLEKMRGEDLQGLTI ESLQNLESTLETGLSDVLGRKSEQI MEQI NGLQQ   154
AEX92969.pro     SPEQPSLDLN. . . LQNSNYARLSKQVVETSRQLRKMRGEDLQGLTI EELQNLEKTLETGLSRVLGRKSEQI MEQI NGLQQ   154

MADS-box_1.pro   REQMLCEANRSLRKRCVQLEETSQANQQVWEANPN. . . . . AMVGYSRQPN. QPQGDEFFHPLECQPTLQ. MGVQ. . PDQN   230
AEX92976.pro     REQMLCEANRSLRKRCVQLEETSQANQQVWEANPN. . . . . AMVGYSRQPN. QPQGDEFFHPLECQPTLQ. MGVQ. . PDQN   230
MADS-box_2.pro   EEQMLQDANRALRSKLQEI GPE. NPLQLSWQNGGGGGGGGGCAGTSAHCNRQPQPEGFFQPLGRDPSSQ. TGFSRVSMDH   234
AEX92975.pro     EEQMLQDANRALRSKLQEI GPE. NPLQLSWQNGGGGGGGGGCAGTSAHCNRQPQPEGFFQPLGRDPSSQ. TGFSRVSMDH   234
MADS-box_6.pro   KFKNSQEAHRNLLRELEMKDEHPAVYGFVDEDPSN. . . . . . YEGALALAN. GGSQMYAFRVXPSQPNLHGMGYG. . . SQD   223
AEX92972.pro     KFKNSQEAHRNLLRELEMKDEHPAVYGFVDEDPSN. . . . . . YEGALALAN. GGSQMYAFRVXPSQPNLHGMGYG. . . SQD   223
MADS-box_5.pro   KGLQLMEENTRLRQQVVDMSQVGKQVVTGLENGSH. . . . . . EEGQSSDSVTNASNSDAPQDYHDSSDTS. LKLC. . LPWN   225
AEX92969.pro     KGLQLMEENTRLRQQVVDMSQVGKQVVTGLENGSH. . . . . . EEGQSSDSVTNASNSDAPQDYHDSSDTS. LKLC. . LPWN   225

MADS-box_1.pro   AGPSVSAFMLG. . . . WL                 243
AEX92976.pro     AGPSVSAFMLG. . . . WL                 243
MADS-box_2.pro   LNSAVTNQNVNSFRHWM                  251
AEX92975.pro     LNSAVTNQNVNSFRHWM                  251
MADS-box_6.pro   LRLA. . . . . . . . . . . . .                227
AEX92972.pro     LRLA. . . . . . . . . . . . .                227
MADS-box_5.pro   LTNTF. . . . . . . . . . . .                230
AEX92969.pro     LTNTF. . . . . . . . . . . .                230
```
